# The usefulness of routine histopathology of bilateral nasal polyps – a systematic review, meta-analysis, and cost evaluation

**DOI:** 10.1186/s40463-015-0100-8

**Published:** 2015-11-04

**Authors:** Jay SM Wong, Stephanie Hoffbauer, David H Yeh, Brian Rotenberg, Michael Gupta, Doron D Sommer

**Affiliations:** Department of Otolaryngology – Head and Neck Surgery, McMaster University Medical Centre, 3V1 Clinic, 1200 Main Street W, Hamilton, ON L8N 3Z5 Canada; Department of Otolaryngology – Head and Neck Surgery, Schulich School of Medicine and Dentistry, Western University, London, ON Canada

**Keywords:** Nasal polyps, Pathology, Cost-effectiveness, Cost evaluation

## Abstract

**Background:**

Controversy regarding the usefulness of routine histopathological examination of bilateral nasal polyps removed during endoscopic sinus surgery to identify occult diagnoses still exists. There is a paucity of high-level evidence in the literature.

**Methods:**

A systematic review and meta-analysis was conducted. Two independent reviewers were used. Pooled proportions and numbers needed to screen were calculated. A cost per life year model was generated based on varying survival benefits and compared to other Canadian screening programs to provide financial context.

**Results:**

Six studies (*n* = 3772 patients) were included. Of the 3772 patients, 3751 had a pre-operative clinical and post-operative pathological diagnosis of inflammatory nasal polyps. Agreement proportion was 99.44 %. There were 18 unexpected benign and three unexpected malignant diagnoses identified. This translated to a proportion of 0.48 and 0.08 % respectively. Number needed to screen was 210 and 1258 respectively. Pooled proportion for expected findings using a random effect model was 0.99 (95 % CI = 0.99–1). Pooled proportion for unexpected benign findings using a random effect model was 0.00522 (95 % CI = 0.00133–0.01). Pooled proportion for unexpected malignant findings using a random effect model was 0.00107 (95 % CI = 0.000147–0.00283). The cost to pick up one unexpected benign diagnosis was $14557.2. The cost to pick up 1 unexpected malignant diagnosis was $87204.56. Cost per quality life year calculated ranged from 3211.83 to $64677.58 based on varying assumptions on the survival benefits of identifying an unexpected malignancy.

**Conclusions:**

Routine pathological examination in screening for neoplasia may be low yield, however, no compelling evidence was found to cease such practice. Surgeons should exercise individual judgment in requesting routine examination.

**Electronic supplementary material:**

The online version of this article (doi:10.1186/s40463-015-0100-8) contains supplementary material, which is available to authorized users.

## Background

Nasal polyps are inflammatory entities found within the nasal cavities [1]. They are considered benign and often present bilaterally. The population prevalence of nasal polyposis is estimated to be approximately 4 % of the population and about 20 % in patients with chronic rhinosinusitis [2, 3]. The main complaint of patients with polyposis is nasal obstruction [3]. The mainstay medical treatment includes the use of steroids and for refractory cases, surgery is often recommended for removal [2]. While not mandated, it has been considered convention to send the removed nasal polyp specimens for routine histopathology to confirm the final pathological diagnosis [4, 5]. Unilateral nasal polyps have higher rates of occult malignancies and as such, there is agreement within the literature to send off all unilateral nasal polyp specimens for pathological analysis to avoid missing an occult diagnosis [1, 6–8]. The practice of routine histopathological analysis is more controversial within the literature for bilateral benign-appearing nasal polyps [1, 5–7]. Various observational studies have noted no discrepancies between pre-operative clinical and post-operative pathological diagnoses of bilateral nasal polyps [1, 6, 7]. These authors concluded that routine examination of bilateral nasal polyps may not be necessary as the yield is low. Furthermore, routine examinations incur costs. As most healthcare systems are financially constrained, the spending accumulated from routine examinations, especially those with low yields, brings into question the justification for such practices. Conversely, other studies have found unexpected diagnoses from routine examinations of bilateral nasal polyps [5, 9]. These findings range from benign entities such as inverted papillomas to malignancies such as squamous cell carcinomas. These results argue for the routine examination of bilateral nasal polyps, despite additional costs, given the clinical and medico-legal implications for missing occult diagnoses.

To date, while there are various individual studies examining this topic, a systematic review has not been completed. As such, the aim of this study was to perform a systematic review and meta-analysis of the literature, comparing pre-operative clinical with post-operative pathological diagnoses of bilateral nasal polyps and associated discrepancies. The study also aimed to provide a financial context of the associated findings.

## Methods

As this was a review and meta-analysis of observational studies, it will be reported according to the guidelines of the Meta-analysis of Observational Studies in Epidemiology Group (MOOSE) [10]. A search of electronic databases was performed with the assistance of a librarian specializing in health sciences database searches. Search terms included “nasal polyp”, “pathology”, “histopathology”, and variations of these terms (Additional file 1). Databases searched were: EMBASE (1974 – June 24, 2014), MEDLINE (1946 – June 24, 2014), Web of Science (1976 – June 24, 2014), and Cochrane Review – EBM reviews off OVID platform (current to June 24, 2014).

Inclusion criteria were: studies comparing the pre-operative clinical diagnosis and post-operative pathological diagnosis of children or adults with bilateral nasal polyps removed during endoscopic sinus surgery and their agreement proportion. Potential study designs included were retrospective chart review, prospective cohort studies, case series, case control studies, systematic reviews, meta-analysis, and randomized controlled trials. Included studies were limited to the English language only. Exclusion criteria were: studies that evaluated exclusively unilateral nasal polyps and associated surgical pathologies or studies that failed to distinguish between unilateral and bilateral nasal polyp specimens and associated surgical pathologies. Case report studies with less than 5 patients were excluded. Non-English studies were excluded as well.

Two independent reviewers (J.W. and S.H.) screened the titles of the search results independently to generate a short-list of articles to be retrieved in full. If it was unclear from the title whether the study was to be included or excluded, it was also incorporated into the short-list for further review. Disagreements were resolved through consensus. Full texts of the short-listed articles were then reviewed separately to ultimately determine eligibility criteria. Again, disagreements were resolved through consensus. The references of the short-listed articles were also reviewed for any studies that could potentially be included. Data from the included studies were extracted independently by the two reviewers using a standardized template determined *a priori*. Data extracted was compared and disagreements resolved through consensus. No contact with authors of the included studies was made. Quality of the individual studies included was appraised using a critical review checklist of the Dutch Cochrane Centre which was proposed by MOOSE [10]. Number needed to screen was calculated by dividing the total number of specimens by the number of unexpected findings as an overall group and benign and malignant findings separately. Meta-analysis was performed by calculating pooled proportions of the weighted occurrence of expected and unexpected histopathological findings using a random effect (DerSimonian Laird) model. Subgroup analysis was performed as well. Heterogeneity between studies was tested using I^2^ statistics. Publication bias was assessed with visual inspection of the funnel plot as well as Egger’s test. StatsDirect software 2.7.9 (StatsDirect Limited, Cheshire, UK) was used.

Costing data was obtained from a previous study [4]. The information was derived from two different academic institutions. At McMaster University, pathologists are salaried and effective hourly wage is estimated at $203. Accounting for an average of 5 min to analyze bilateral nasal polyp specimens, the pathological interpretation cost would be $16.90 (5 min × $3.38 per minute). The technical preparation cost associated is $33.08. Combining the pathological and technical costs together, the total would be $49.98 per bilateral specimen analyzed. At Western University, the pathologist interpretation cost is $48.65 per bilateral specimen. Technical and report preparation amount to $40. This brings the total to $88.65 per bilateral specimen analyzed. To improve generalizability, an average of the two methods of remuneration was calculated. The average was C$69.32.

The primary analysis was to perform a systematic review of the literature to determine the number needed to screen and associated costs to pick up one unexpected finding. Unfortunately, these results are difficult to compare to other studies and are difficult for health care decision makers to use when determining the adoption of various clinical practices. To derive a result that could better compare routine screening of nasal polyposis to other forms of routine screening and health expenditure, a secondary analysis was performed to calculate the cost per life year gained from the practice. To perform this secondary analysis, assumptions were necessary. Chief among these assumptions was the mortality benefit imparted by detection of the unexpected diagnoses. There was no published literature to guide assumptions in this regard. As a consequence, any assumptions stood on very shaky ground. To reduce bias in the analysis, four models were created, each assumed a different mortality benefit from detection on histopathology. The first scenario used published AJCC five-year survival rates for sinonasal malignancy and assumed detection improved survival from the worst stage (Stage IV) to the best stage (Stage I) [11]. The improved mortality rate in this scenario was 28 %. The rationale behind this assumption was that detected disease allowed earlier recognition and prompt treatment that would likely be successful, whereas, patients that go undetected will present with advanced disease. A second scenario was created that assumed an even greater improved survival of 80 % and a third scenario considered a reduced improvement in survival of only 5 %. These numbers were chosen in a deterministic fashion. A final scenario considered 100 % improvement in survival. In all four models, only unexpected cases of malignancy were considered. Benign pathologies were excluded as assumptions regarding mortality benefits of early detection of benign disease added an undesirable layer of complexity and uncertainty to the analysis.

To generate the variables of the model, proportions and costs were derived from the primary analysis and the assumptions in each model. A standard life expectancy of 81.24 years was used based on the most recent data from Statistics Canada. A decision tree model was created. The Ministry of Health payer perspective was taken. A cost per life year was calculated. Each year lived past the diagnosis of malignancy was assumed to have a utility of 1. For each model, a base case result was obtained. Next, the models were subjected to a probabilistic sensitivity analysis. Proportions were varied on a beta distribution, and costs and patient ages on a gamma distribution. Where standard errors were unknown, a conservative estimate of 25 % was made. A discount rate of 5 % was used for future benefits. Each model underwent 1000 simulations and a mean result was recorded. The results from the generated model were then compared to another cancer screening program in Canada, specifically, colorectal screening to provide context for the calculated values.

## Results

**Fig. 1:**
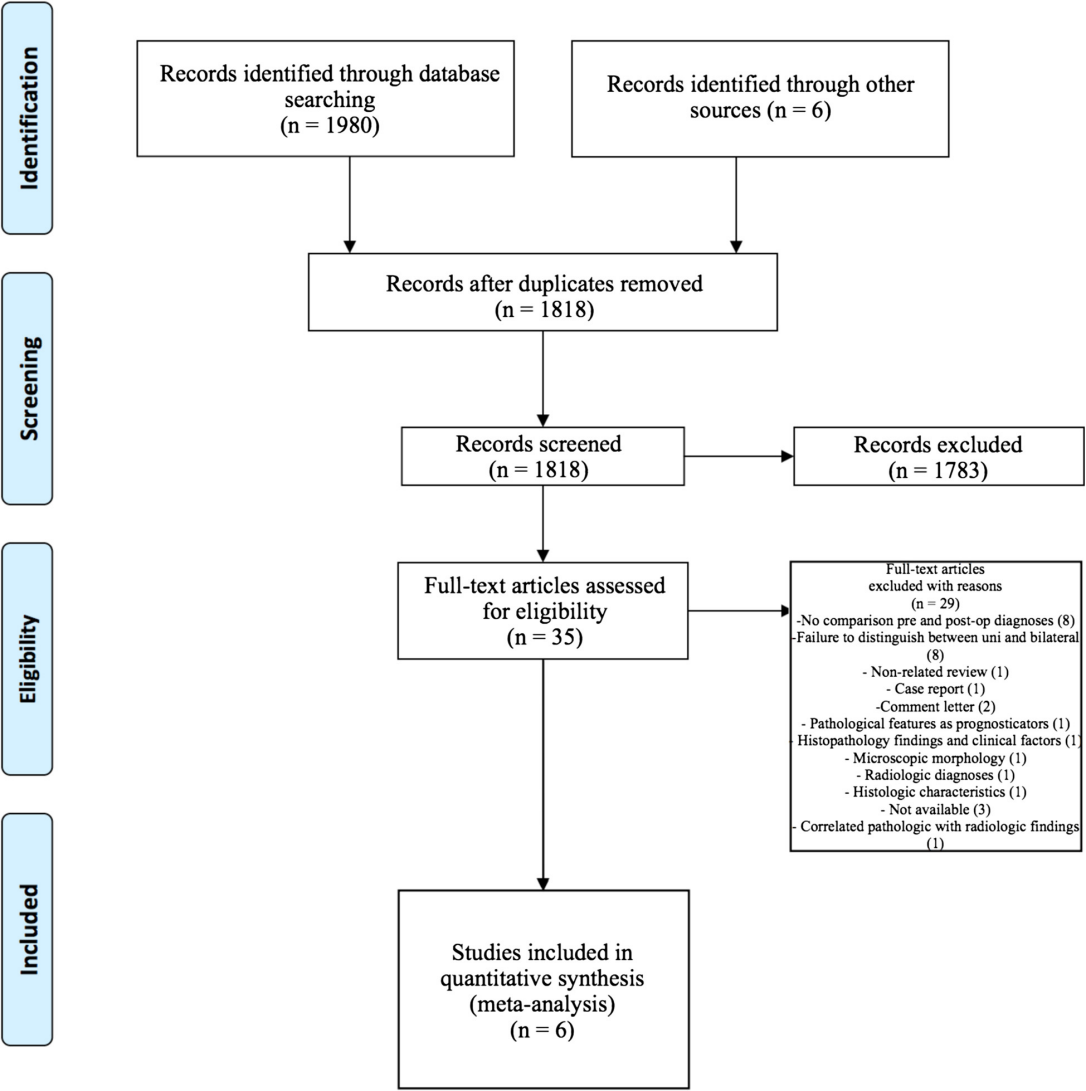
PRISMA chart of systematic review process. The number of studies at each stage of the systematic review process is highlighted in parentheses. For full text articles that were excluded, reasons given in diagram

**Fig. 2:**
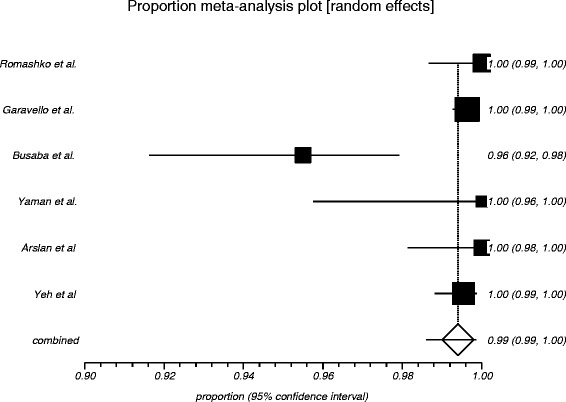
Graphical representation of meta-analysis plot using a random effects model for expected findings

**Fig. 3:**
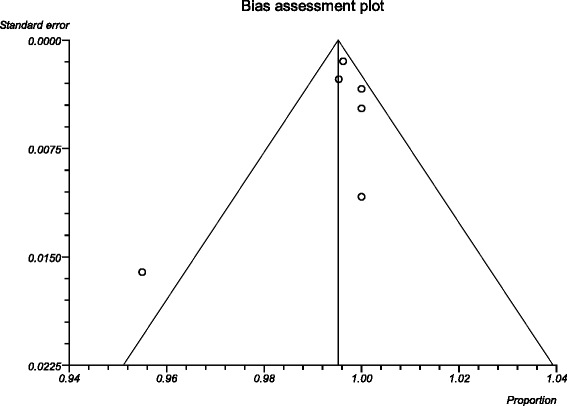
Funnel plot of publication bias. The symmetry of the funnel plot indicates minimal publication bias

**Fig. 4:**
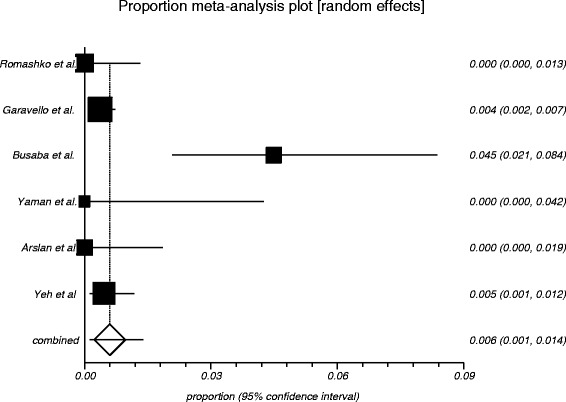
Graphical representation of meta-analysis plot using a random effects model for overall unexpected findings

**Fig. 5:**
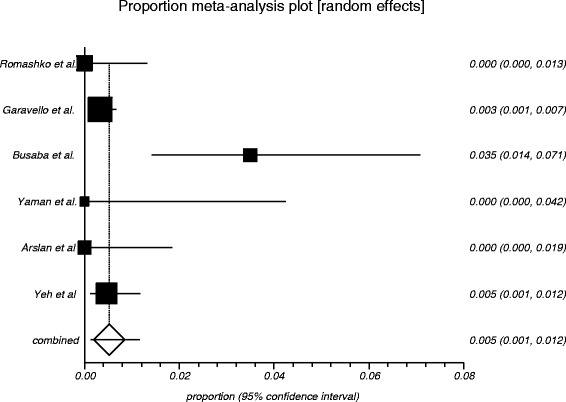
Graphical representation of meta-analysis plot using a random effects model for unexpected benign findings

**Fig. 6:**
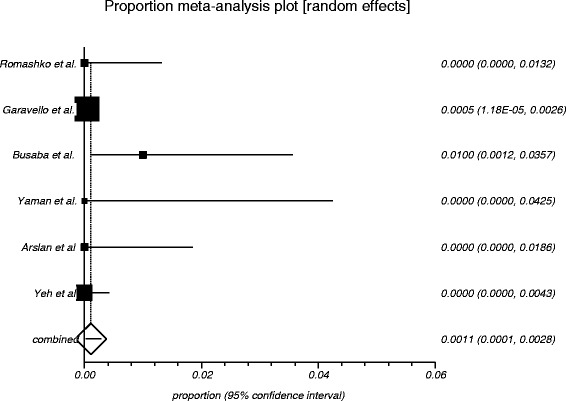
Graphical representation of meta-analysis plot using a random effects model for unexpected malignant findings

**Fig. 7:**
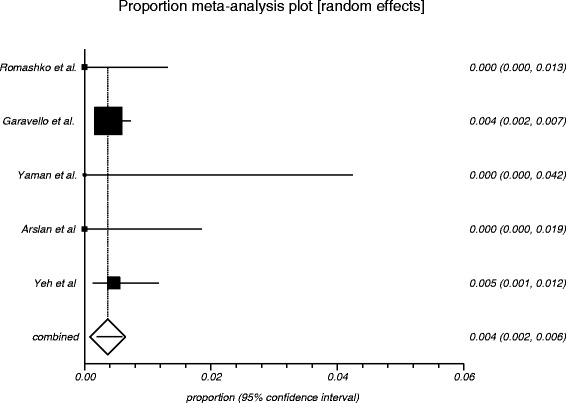
Graphical representation of subgroup meta-analysis plot using a random effects model for overall unexpected findings

**Fig. 8:**
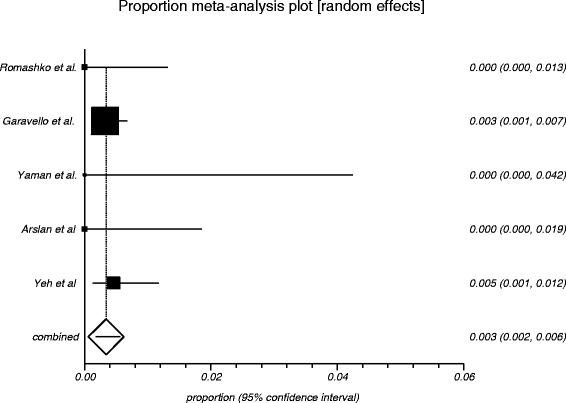
Graphical representation of subgroup meta-analysis plot using a random effects model for unexpected benign findings

**Fig. 9:**
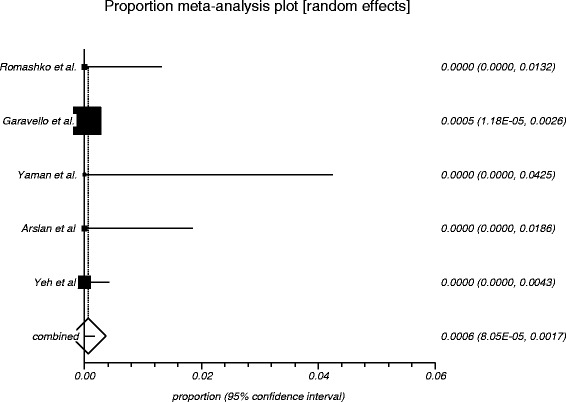
Graphical representation of subgroup meta-analysis plot using a random effects model for unexpected malignant findings

**Fig. 10:**
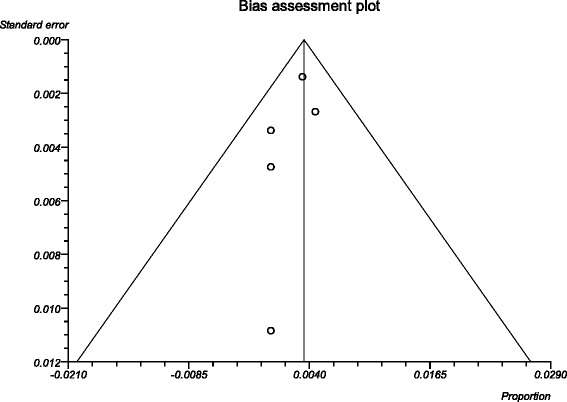
Funnel plot of publication bias (subgroup analysis). The symmetry of the funnel plot indicates minimal publication bias

The process from initial search to final studies included is outlined in a flow diagram [12] (Fig. 1). Six studies ultimately met inclusion criteria for this systematic review [1, 4–7, 9]. All included studies were observational studies. Quality of the individual studies was appraised using a critical review checklist as proposed by MOOSE [10] (Table 1). Further details for each of the included studies are outlined (Table 2). In regards to age and gender of the studied populations, the mean and standard deviation reported in various studies was an overall value including both patients with unilateral and bilateral nasal polyps. Therefore it was hard to determine the true mean age and standard deviation as well as gender characteristics for bilateral nasal polyp patients alone. Garavello et al. did report a mean age with standard deviation (44.9 +/− 10.2 years) specifically for bilateral nasal polyp patients [5]. This bilateral nasal polyp population was comprised of 1244 males and 903 females. In total, there were 3772 bilateral nasal polyp specimens. Of these, 3751 (99.44 %) had pre-operative clinical and post-operative pathological diagnoses of inflammatory nasal polyps. There were 21 (0.56 %) unexpected post-operative pathological results – 18 (0.48 %) benign and 3 (0.08 %) malignant. Breakdown of the unexpected diagnoses is shown as a group and by individual studies (Table 3, Table 4). Number needed to screen (NNS) to pick up 1 overall unexpected finding was 180. NNS for an unexpected benign finding was 210 and 1258 for an unexpected malignant finding. The pooled proportion for expected findings using a random effect model was 0.99 (95 % CI = 0.99–1) (Fig. 2). The I^2^ inconsistency statistic was 76.3 % (95 % CI = 29.9–87.7 %) indicating a considerable degree of heterogeneity. The funnel plot indicated minimal publication bias (Fig. 3). Pooled proportion for overall unexpected findings using a random effect model was 0.00599 (95 % CI = 0.00133–0.01) (Fig. 4). Pooled proportion for unexpected benign findings using a random effect model was 0.00522 (95 % CI = 0.00133–0.01) (Fig. 5). Pooled proportion for unexpected malignant findings using a random effect model was 0.00107 (95 % CI = 0.000147–0.00283) (Fig. 6). The I^2^ inconsistency statistic was 19.9 % (95 % CI = 0–68.3 %), indicating low heterogeneity. Examination of the funnel plots revealed minimal publication bias. The cost to pick up one unexpected diagnosis was $12477.6 (180 × $69.32). The cost to pick up 1 unexpected benign diagnosis was $14557.2 (210 × $69.32). The cost to pick up 1 unexpected malignant diagnosis was $87204.56 (1258 × $69.32). A subgroup analysis of pooled proportions was performed excluding Busaba et al. paper. Pooled proportion for overall unexpected findings using a random effect model was 0.00370 (95 % CI = 0.00197–0.00595) (Fig. 7). Pooled proportion for unexpected benign findings using a random effect model was 0.00342 (95 % CI = 0.00178–0.00560) (Fig. 8). Pooled proportion for unexpected malignant findings using a random effect model was 0.000643 (95 % CI = 0.0000805–0.00174) (Fig. 9). The I^2^ inconsistency statistic was 0 % (95 % CI = 0–64.1 %) indicating a low degree of heterogeneity. Funnel plot indicated minimal publication bias (Fig. 10).

**Table 1:** Assessment of quality of included studies

Questions	Yeh et al. 2014	Yaman et al. 2011	Arslan et al. 2010	Romashko et al. 2005	Busaba et al. 2005	Garavello et al. 2005
Clear definition of study population?	Yes	Yes	Yes	Yes	Yes	Yes
Clear definition of outcomes and outcome assessment?	Yes	Yes	Yes	Yes	Yes	Yes
Independent assessment of outcome parameters?	No	No	No	No	No	No
Sufficient duration of follow-up?	N/A	N/A	N/A	N/A	N/A	N/A
No selective loss during follow-up?	N/A	N/A	N/A	N/A	N/A	N/A
Important confounders and prognostic factors identified?	No	No	No	Yes	Yes	Yes

*N/A* not applicable

**Table 2:** Overview of included studies for systematic review

Study:	Study design:	Time period:	Male to female ratio:	Total number of bilateral specimens:	Number in agreement (percentage):	Number not in agreement (percentage):	Number not in agreement – benign (percentage):	Number not in agreement – malignant (percentage):
Yeh et al. 2014	Prospective chart review	2007–2013	N/A	866	862 (99.54 %)	4 (0.46 %)	4 (0.46 %)	0 (0 %)
Yaman et al. 2011	Retrospective chart review	2005–2010	N/A	85	85 (100 %)	0 (0 %)	N/A	N/A
Arslan et al. 2010	Retrospective chart review	2000–2009	N/A	197	197 (100 %)	0 (0 %)	N/A	N/A
Romashko et al. 2005	Retrospective chart review	1986–2003	N/A	277	277 (0 %)	0 (0 %)	N/A	N/A
Busaba et al. 2005	Retrospective chart review	N/A	N/A	200	191 (95.5 %)	9 (4.5 %)	7 (3.5 %)	2 (1 %)
Garavello et al. 2005	Retrospective chart review	1991–2004	1244:903	2147	2139 (99.62 %)	8 (0.37 %)	7 (0.33 %)	1 (0.05 %)

*N/A* not available

**Table 3:** Breakdown of unexpected post-operative pathological diagnoses

Unexpected Benign Diagnoses (*N* = 18):	Number of:
Inverted papilloma	16
Chronic invasive fungal sinusitis	1
Sinonasal sarcoidosis	1
Unexpected Malignant Diagnoses (*N* = 3):	Number of:
Adenocarcinoma	2
Squamous cell carcinoma	1

**Table 4:** Breakdown of unexpected pathological diagnoses by individual studies

Studies:	Unexpected pathological diagnosis (benign):	Unexpected pathological diagnosis (malignant):
Yeh et al. 2014	4 inverted papilloma	None
Yaman et al. 2011	None	None
Arslan et al. 2010	None	None
Romashko et al. 2005	None	None
Busaba et al. 2005	5 inverted papilloma	1 adenocarcinoma
	1 chronic invasive fungal sinusitis	1 squamous cell carcinoma
	1 sinonasal sarcoidosis	
Garavello et al. 2005	7 inverted papilloma	1 adenocarcinoma

The cost per quality life year model based on varying survival benefits from routine examination is shown in a table format (Table 5). The most pessimistic model, assuming a survival benefit of only 5 %, had a base case result of $40075.90 per life year and a probabilistic result of $64677.58 per life year. The cost was least expensive at $2003.80 (base case) and $3211.83 (probabilistic case) per quality life year assuming a survival benefit of 100 %.

**Table 5:** Cost per life year model based on varying survival benefits from routine examination

Model (% survival improvement)	Base Case (2015 C$/life year)	Probabilistic (2015 C$/life year)
28 %	7156.41	11759.55
80 %	2504.74	3919.95
100 %	2003.80	3211.83
5 %	40075.90	64677.58

## Discussion

The practice of sending all non-suspect bilateral nasal polyp specimens for routine histopathology in search of unexpected diagnoses remains to be a controversial topic. The controversy can be broken down into two aspects, alteration of management and cost. Various authors had shown through their studies that there were no discrepancies between clinical and pathological diagnoses of non-suspect bilateral inflammatory nasal polyps [1, 6, 7]. As such, these authors concluded that routine examination of benign-appearing bilateral nasal polyps is perhaps unnecessary, as the findings do not alter management and incur additional financial cost. Conversely, other authors have recommended routine examination of all bilateral nasal polyps as their study series had shown unexpected findings, both benign and malignant [5, 9]. These unexpected findings arguably could have outcome-altering and medico-legal ramifications. As such, it would seem justified to perform routine examinations even though the pickup rate is low and additional cost is incurred.

For this current meta-analysis, based on eligible studies (*N* = 6), the pooled proportion for unexpected overall findings was 0.00599. For unexpected benign findings it was 0.00522 and 0.00107 for unexpected malignant findings. Reviewing the individual studies included, Busaba et al. study was noted to be a potential outlier. To ensure that this did not skew the results significantly and affecting subsequent calculations, a subgroup meta-analysis of the other five studies was performed. The pooled proportions from the subgroup analysis were not significantly different from the corresponding values from the general meta-analysis. Furthermore, the unexpected benign and malignant proportions from the general and subgroup meta-analyses were within each other’s confidence intervals. In other words, they were within the error of each other. This provided reassurance that including the potential outlier did not significantly skew the results and subsequent calculations.

Garavello’s study was the largest of all the studies included, examining over 2000 bilateral nasal polyp specimens. The study identified seven inverted papillomas and one adenocarcinoma. The authors concluded that occult pathology is a rare but possible event. Busaba’s study identified nine unexpected diagnoses from a total of 200 bilateral specimens (4.5 %). Of the nine unexpected findings, seven were benign and two were malignant (Table 4). Five of the seven benign findings were inverted papillomas. Three of the patients underwent revision surgeries while the other two were followed serially by nasal endoscopy. No recurrence was noted in all five patients with follow-up ranging from 2 to 5 years. The patient with squamous cell carcinoma subsequently underwent revision surgery followed by radiation therapy. The patient has remained disease-free for 3 years. The patient diagnosed with adenocarcinoma was treated with proton beam radiation therapy without additional surgery and has remained disease-free for 5 years. Given these findings, Busaba and colleagues recommended submitting all bilateral specimens for routine examination, ideally the full surgical specimen rather than samples.

Grouping all six studies together, there were 21 unexpected diagnoses (0.56 %), specifically, 18 benign (0.48 %) and three malignant (0.08 %) from a total of 3772 bilateral specimens. Of these, 16 were unexpected diagnoses of inverted papillomas, which are considered benign albeit locally aggressive. As reported, three cases underwent revision surgery while six others were only followed with serial nasal endoscopy without additional surgery. No evidence of recurrence was noted through long-term follow-up of these patients. Specific information regarding the other cases was not provided. In another study that did not meet inclusion criteria, two cases of bilateral nasal polyps yielded an unexpected finding of inverted papilloma [13]. Similarly, these cases did not require revision surgery and were followed up long-term with no evidence of recurrence. As brought forth in Romashko’s discussion, one study analyzed 33 polypectomy cases pathologically and identified foci of dysplasia and malignancy [14]. No focus was larger than 0.1 cm and no patient had evidence of recurrence with a mean follow-up of 6 years. Foci of such sizes might escape clinical detection but were identified on pathological exam. Given that there were no cases of recurrence for those patients followed with serial endoscopy from this review, this would suggest that perhaps the initial surgery was curative and patient outcome would not change if routine analysis was not performed. It is important to note that this premise of “cure” from initial surgery is based on a very small number of cases. It does however suggest the importance of regular follow-up in this population. Conversely, given the fact that there is an 8–10 % malignancy transformation rate for inverted papillomas, a contrary argument for routine examination can be made. This is on the basis that there are implications for subsequent treatment and surgery to minimize chances of recurrence or malignant transformation. A similar argument can be made as well for early pick-up of other benign entities such as sarcoidosis and granulomatosis with polyangiitis as there are implications for further work-up and early intervention that can change disease course. As reiterated within the literature, whether or not sending the specimens for routine analysis truly makes a difference in outcome for both malignant and benign entities can likely only be answered by a large prospective study.

In regards to unexpected malignant diagnoses, there were three cases noted from the review. Information regarding outcome and follow-up was only available for two of the cases [9]. In both cases, there was further treatment and long-term follow-up noted no recurrence. Given that there were unexpected malignant findings, one would argue for routine examination regardless of cost. This study aimed to provide new information by attempting to put the cost aspect into perspective. Such information is currently sparse within the literature. Data derived from a study evaluating the cost-effectiveness of colorectal screening in Canada was used for comparison purposes [15]. The incremental cost-effectiveness of low-sensitivity guaiac fecal occult blood test performed annually, fecal immunochemical test performed annually, and colonoscopy performed every 10 years, was 9159, 611, and $6133 per quality-adjusted life year respectively. Overall, the cost per quality life year from routine screening of bilateral nasal polyps appeared to be more expensive than colorectal screening. Despite this, for three of the scenarios (28, 80, and 100 % survival benefit), the cost falls well below the conventionally accepted willingness to pay cost of $50000 per life year. For the scenario of 5 % survival benefit, the cost was $64677.58, which did not differ drastically from the conventionally accepted value for willingness to pay. Such findings would suggest the practice of routine screening to be justifiable. It is important to keep in mind that the model was based on various assumptions and some perhaps considered not universally applicable. The purpose of the model was meant to generate further discussion. Ultimately, without a prospective study, it would be impossible to definitively determine the benefits for patients who are picked up due to routine examination in terms of outcomes compared to those who are missed by not sending for analyses. Currently, this review found no compelling evidence to change the practice of routine histopathologic examination of bilateral polyp specimens. As such, judgment must be utilized by the individual surgeon to determine the need to request for routine examination.

Limitations of this systematic review include the fact that it is based on a small number of studies. Furthermore, these studies were all observational studies, and as such, prone to selection bias, confounding, and the use of different selection criteria and presence of varying referral patterns among studies. The method of obtaining and submitting the surgical specimens also varied among studies. While not explicitly stated in every study, methods of obtaining specimens ranged from selective samples of whole polyps to microdebrider specimens. These different ways of obtaining the samples might result in varying representations of the specimens, potentially leading to false-negative results, ultimately affecting the true rate of pickup. The economic evaluation was limited primarily by the lack of evidence on the mortality benefit of identification of unexpected malignancies. Other limitations included the exclusion of the benefit of identifying unexpected, non-malignant diagnoses and the assumption of perfect utility in patients surviving sinonasal malignancies.

Aside from picking up occult benign and malignant diagnoses, there are other reasons proposed to submit for routine analysis. The inflammatory profile of chronic rhinosinusitis (CRS) can be subdivided into predominantly eosinophilic and non-eosinophilic/neutrophilic [16]. The disease process for the two groups is different. Eosinophilic chronic rhinosinusitis (ECRS) is often associated with greater symptom severity and poorer outcome [17–19]. ECRS requires more aggressive treatment such as the use of stronger systemic and topical corticosteroids and is often refractory to surgical management. It is also usually associated with polyps, asthma, high serum eosinophilia, aspirin sensitivity, and immunoglobulin E (IgE). Unfortunately, these findings are not consistently seen and cannot be relied upon to diagnose someone with ECRS. These studies suggested that high tissue eosinophilia from histopathological analysis of nasal polyps can serve as a marker for the diagnosis of ECRS. This in-turn can provide prognostic information and help guide specific treatment approach to optimize outcomes for ECRS patients [17]. These studies suggest that routine analysis could indeed be helpful in this regard. Notably, the pathological analysis in these studies is significantly more detailed and includes, amongst others, eosinophil count, basement membrane thickening/measurement and more detailed mucin reporting and as such, may incur higher pathological analysis-related costs than those reported in our study. Further evaluation and standardization of this indication for nasal polyp pathological evaluation is warranted.

## Conclusion

Contrasting studies exist in regards to noted discrepancies between pre-operative clinical and post-operative pathological diagnoses of bilateral nasal polyps. Pooled data from this review showed that while the percentage was very low, there were unexpected benign and malignant pathological diagnoses. This review, through its various analyses, highlighted that while routine pathological examination screening for neoplasia may be of low yield, no compelling evidence was found to cease such practice. The surgeon should exercise individual judgment in requesting pathological assessment. To truly answer the usefulness of routine examination, large prospective studies would be required.

## Additional file

Additional file 1:
**MEDLINE search strategy.** An example of the search strategy used for this systematic review. The MEDLINE search strategy is presented here. (PDF 119 kb)
