# Supplementary material for: The usefulness of routine histopathology of bilateral nasal polyps – a systematic review, meta-analysis, and cost evaluation
Source: J Otolaryngol Head Neck Surg. 2015 Nov 4;44:46. doi: 10.1186/s40463-015-0100-8 (PMC4632485; doi:10.1186/s40463-015-0100-8)
Supplement: Additional file 1: — MEDLINE search strategy. An example of the search strategy used for this systematic review. The MEDLINE search strategy is presented here. (PDF 119 kb) [file 40463_2015_100_MOESM1_ESM.pdf]

Set	Search Statement
1.	bilateral nasal polyp*.mp.
2.	Nasal Polyps/
3.	Nose Polyp*.mp.
4.	nasal polyp*.mp.
5.	rhinosinusal polyp*.ti,ab.
6.	nasosinusal polyp*.ti,ab.
7.	or/2-6
8.	histopatholog*.mp.
9.	pathology/ or pathology, clinical/ or pathology, surgical/
10.	pathology.mp.
11.	surgery pathology.ti,ab.
12.	surgical pathology.ti,ab.
13.	clinical pathology.ti,ab.
14.	pathological.ti,ab.
15.	or/8-14
16.	7 and 15
17.	1 or 16
18.	limit 17 to english language
